# Omental Infarction with Acute Appendicitis in an Overweight Young Female: A Rare Presentation

**DOI:** 10.1155/2019/8053931

**Published:** 2019-04-07

**Authors:** Vishnu R. Mani, Shantanu Razdan, Tonny Orach, Aleksandr Kalabin, Rinil Patel, Ali Elsaadi, Kiyoe Sullivan, Federico Gattorno

**Affiliations:** ^1^Department of Surgery, Columbia University College of Physicians and Surgeons at Harlem Hospital Center, New York, NY 10037, USA; ^2^Department of Surgery, Woodhull Medical Center, Brooklyn, NY 11206, USA; ^3^Department of Pathology, Woodhull Medical Center, Brooklyn, NY 11206, USA

## Abstract

Omental infarction is an uncommon cause of acute abdomen but one that clinically mimics more serious and common causes of acute abdomen like appendicitis and cholecystitis. Historically, it was diagnosed only intraoperatively during surgery for presumed appendicitis or other causes of acute abdomen. But with the increase in the use of imaging, especially abdominal computed tomography (CT) scan in the work-up for acute abdomen, more cases of omental infarction are being diagnosed preoperatively. This has also led to the observation that omental infarction is a self-limiting condition which can be managed conservatively. Currently, conservative management and surgery are the only treatment options for omental infarction with no consensus as to the best treatment modality. Having a patient with both acute appendicitis and omental infarction simultaneously is extremely rare with only two reported cases in the literature thus far. Here, we present a 10-year-old obese female who presented to our hospital with acute abdomen and was found to have acute appendicitis and omental infarction. The patient underwent laparoscopic appendectomy and resection of the infarcted omentum and had uneventful recovery and was discharged on the second postoperative day. In this report, we present a review of current literature on omental infarction and highlight the importance of imaging especially abdominal CT scan in the nonoperative diagnosis and treatment of omental infarction.

## 1. Introduction

Acute appendicitis is a very common cause of acute abdomen with children and adolescents having the highest incidence [1]. Omental infarction, however, is a rare cause of acute abdomen but one which can be difficult to differentiate from acute appendicitis on clinical grounds alone [2, 3]. Having both acute appendicitis and omental infarction is extremely infrequent with only two cases reported in the literature: one in an adult female [4] and the other in a 7-year-old girl [5]. Here, we present a 10-year-old obese girl who was managed for acute appendicitis and omental infarction which were initially missed on abdominal and pelvic CT scan.

## 2. Case Presentation

A 10-year-old overweight Hispanic female (BMI > 28) complaining of abdominal pain for 2 days was brought to our emergency department (ED) by her mother. The pain reportedly started in the periumbilical area and later localized to the right lower quadrant. She also reported left lower quadrant pain, anorexia, and nausea but denied vomiting. She had no fever. Upon arrival to the emergency department, her vital signs were within normal limits for her age, but physical exam revealed generalized lower abdominal tenderness with rebound and guarding.

**Figure 1:**
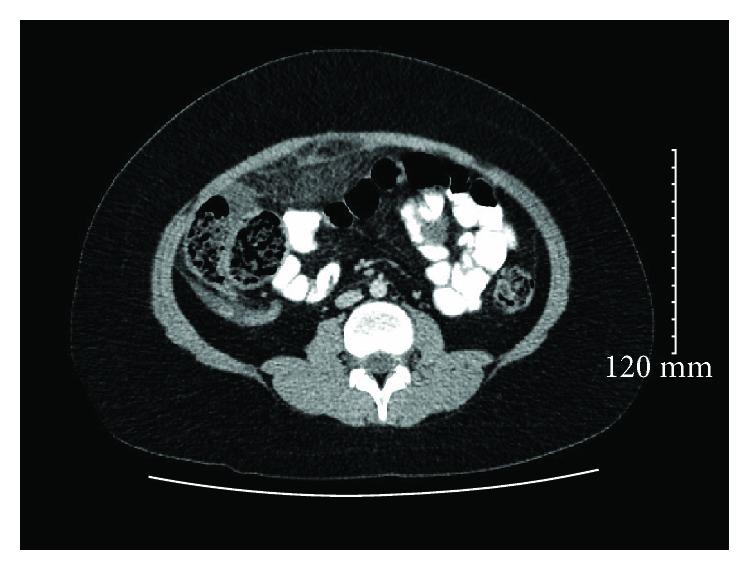
Axial CT demonstrating a small amount of free fluid around the omentum.

**Figure 2:**
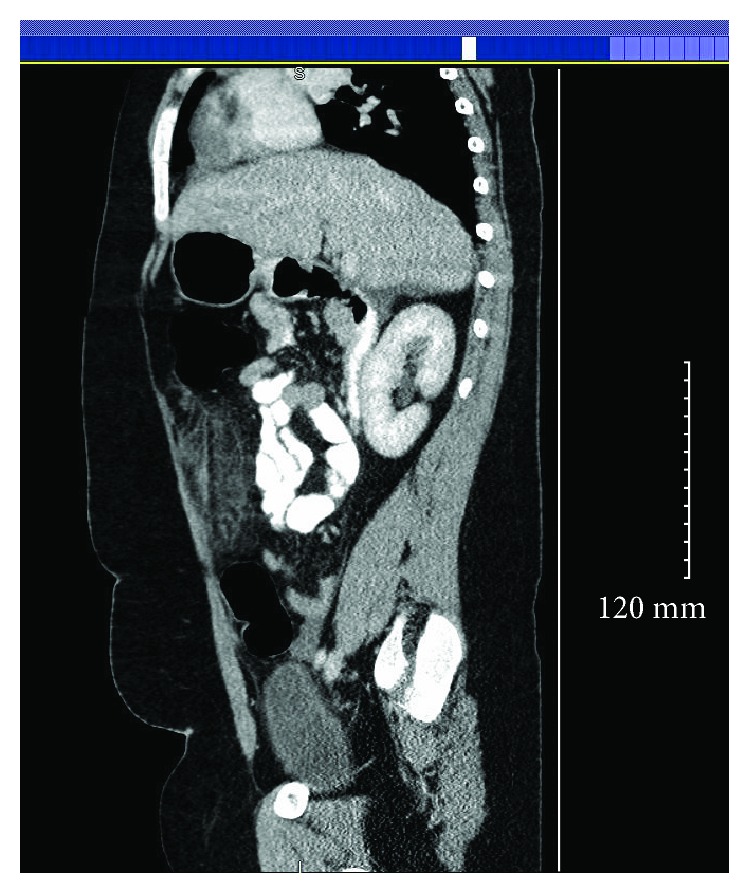
Sagittal CT indicating omental inflammation and fat stranding.

**Figure 3:**
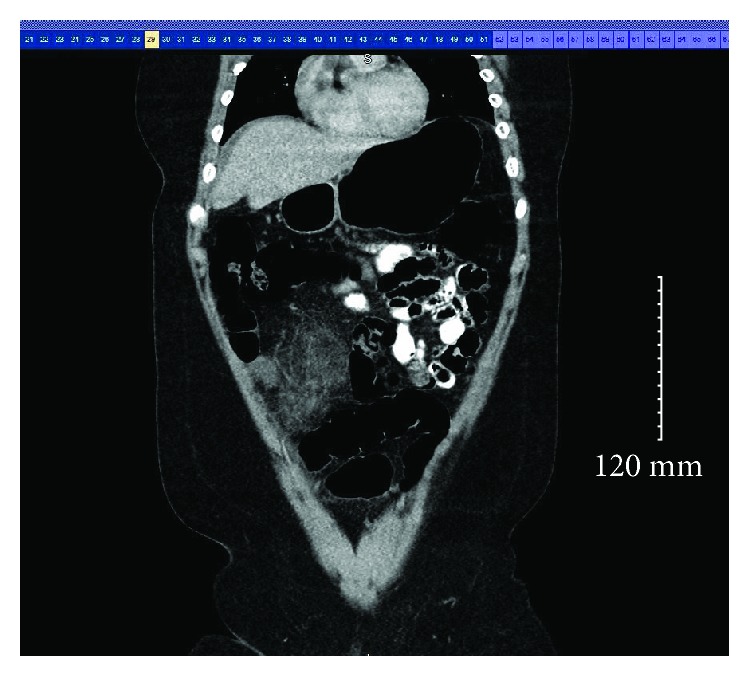
Coronal CT having signs of omental infarction.

Her laboratory investigation was positive for elevated white cell count of 12.10 per microliter of blood with a left shift. The rest of her laboratory results were within normal limits. Abdominal and pelvic CT scan were done which showed hyperemic appendix and hazy anterior mesentery and a small amount of free fluid (Figures 1–3).

**Figure 4:**
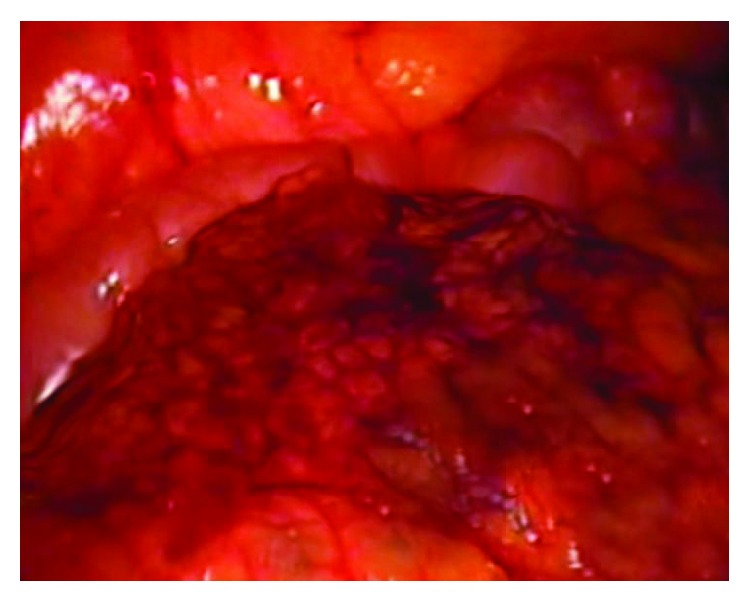
Gross image showing hemorrhagic infarction and necrosis of the omentum.

**Figure 5:**
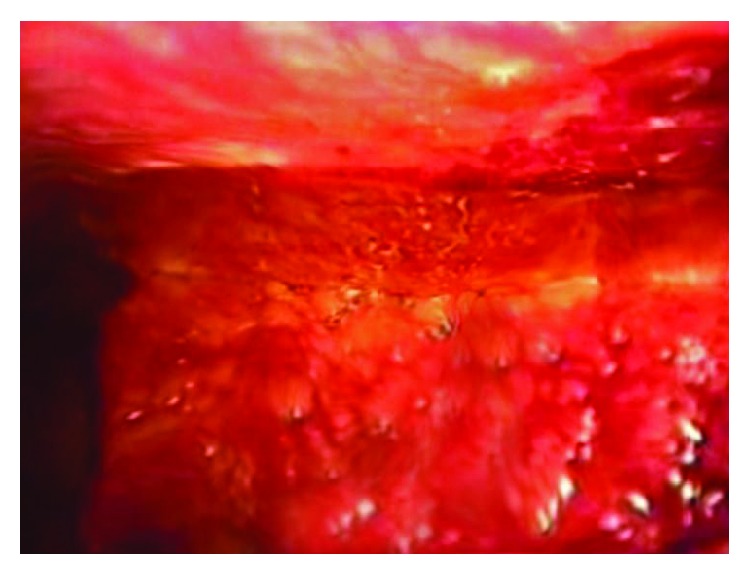
Gross image with hemorrhagic, infarcted omentum adhered to the anterior abdominal wall.

Based on the history, physical exam, and CT scan findings, a diagnosis of acute appendicitis was made and the patient was taken to the operating room for laparoscopic appendectomy. Intraoperatively, a tortuous retrocecal appendix with free fluid was found, and appendectomy was performed without any complications. After performing the appendectomy, the omentum was noted to be adherent to the anterior abdominal wall and on careful examination, it was noted to be hemorrhagic and necrotic as shown in Figures 4 and 5. The necrotic omentum was resected and sent for histopathological examination. Although the infarcted omentum was mostly in the midline anterior abdominal wall, we did not have the necessity to place additional ports and were able to successfully resect the omentum with standard laparoscopic appendectomy port placements (umbilical port, left iliac fossa port, and suprapubic port). The patient had an uneventful postoperative course and was discharged on the second operative day.

**Figure 6:**
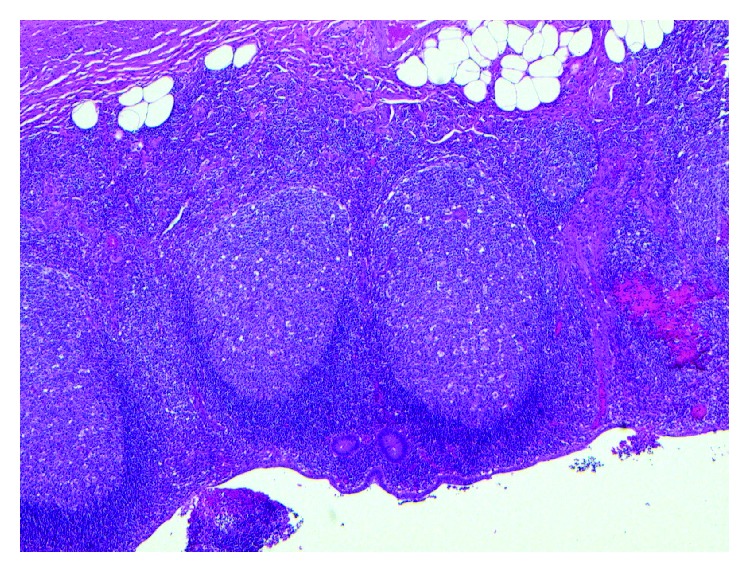
Peyer's patches in the appendix with an area of hemorrhage.

**Figure 7:**
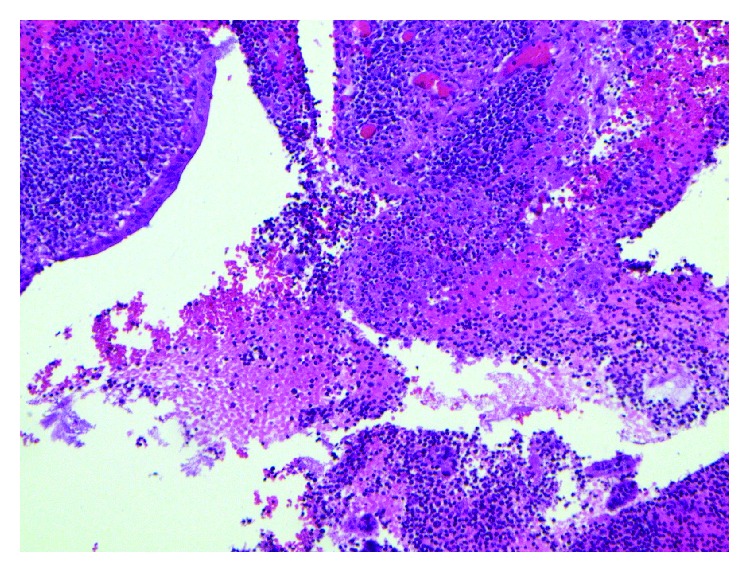
Appendix with focal mucosal hemorrhage.

**Figure 8:**
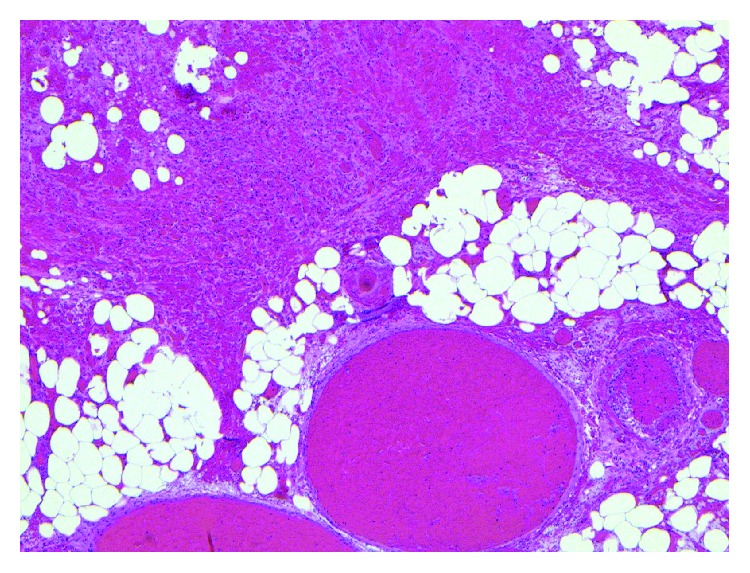
Omentum–vascular congestion and hemorrhage.

**Figure 9:**
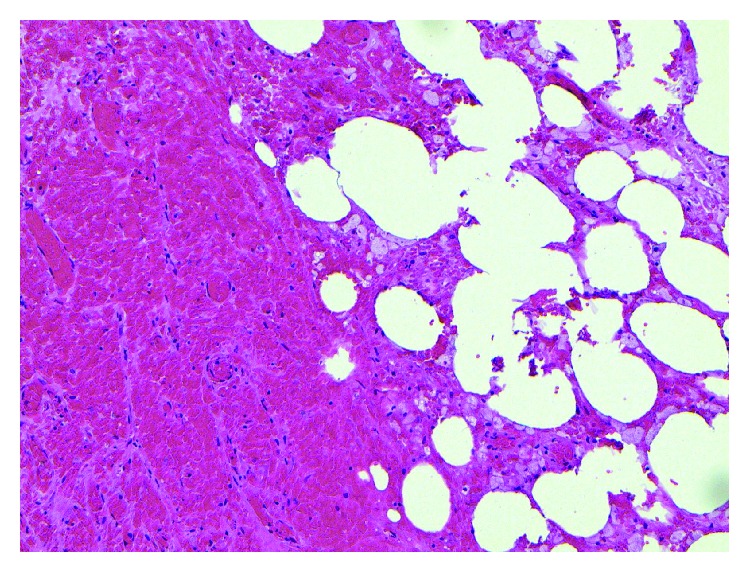
Omentum–hemorrhage and fat necrosis.

**Figure 10:**
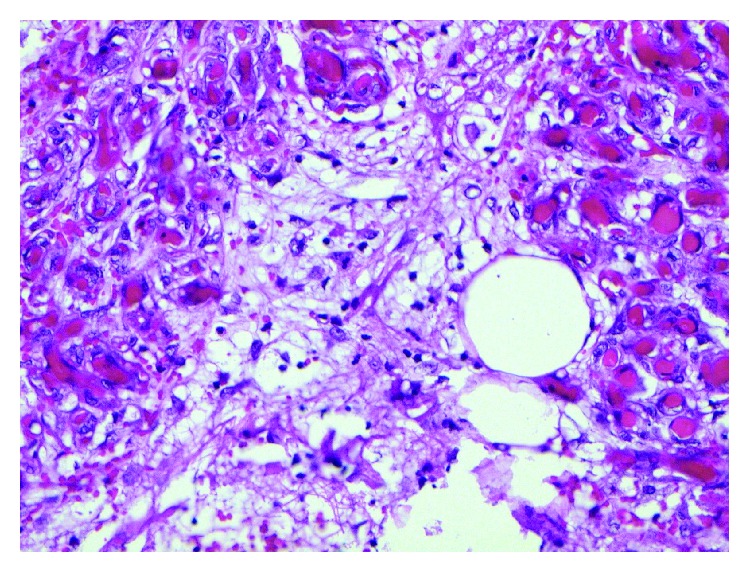
Omentum–focal granulation tissue formation.

**Figure 11:**
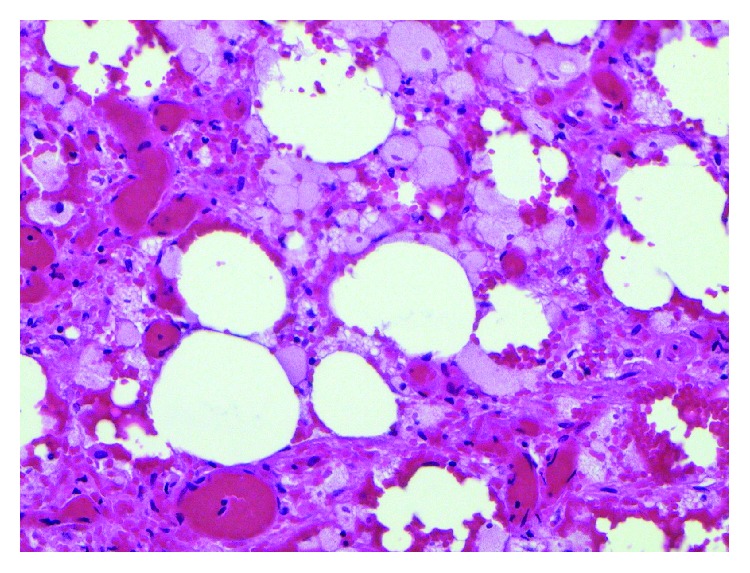
Omentum–fat necrosis.

Histopathological exam of the appendix revealed focal superficial acute mucositis and recent hemorrhage suggesting early acute appendicitis. Examination of the omental mass showed fragments of adipose tissue with hemorrhage, fat necrosis, and granulation tissue formation consistent with omental infarction (Figures 6–11).

## 3. Discussion

Omental infarction is a rare cause of acute abdomen with reported incidence being less than 4 per 1000 cases of appendicitis [6]. It usually presents as right-sided abdominal pain although seldomly causing left-sided abdominal pain and even epigastric pain [7, 8]. The dominion of right-sided abdominal pain in omental infarction has been attributed to right segmental infarction as a result of the tenuous blood vessels in this part of the omentum as well as its longer size and higher mobility in comparison to the left side which subjects it to torsion [2, 6, 7, 9]. Obesity as seen in our patient is a known risk factor for omental infarction. The theory behind this is that fat accumulation within the omentum occludes blood supply to the distal parts of the omentum in addition to making it more susceptible to torsion [2]. Other risk factors for omental infarction are polycythemia, hypercoagulability, and vasculitides plus other conditions which predispose to torsion such as trauma, sudden body movements, coughing, heavy food intake, and hyperperistalsis [2].

Despite being rare, more cases of omental infarction are being reported in recent literature due to the increasing availability and use of imaging modalities especially CT scan in the work-up for acute abdomen [2, 7] leading to more cases being diagnosed preoperatively unlike in the past where only 0.6% to 4.8% of omental infarction were diagnosed nonoperatively [6]. However, our case was diagnosed intraoperatively despite doing abdominal and pelvic CT scan. A number of factors might have contributed to the missed diagnosis. Firstly, our patient presented with clinical features suggestive of acute appendicitis and CT scan confirmed this. The presence of a more serious pathology rightfully took all the attention although the same scan had features suggestive of omental infarction as well. And the radiologist did not commit to a simultaneous diagnosis of omental infarction although retrospectively during a radiology meeting, features of omental infarction were obvious and detected on the CT scan. This highlights the importance of experience for both the radiologist and the physician and having a high index of suspicion in order to diagnose rare conditions like omental infarction.

Radiological features of omental infarction are not straightforward as seen in our case and also as reported by Itenberg et al. [6]. The two most common imaging modalities used are abdominal ultrasound and CT scans. Sonographic features suggestive of omental infarction are a noncompressible hyperechoic ovoid mass, while CT scan findings include the classic “whirl sign” (whirling patterns of fat and vessels in the omentum) and concentric linear strands or caking of the omental fat [2, 6]. Additionally, free peritoneal fluid is seen as was for our patient [10]. However, none of these features except for the “whirl sign” is specific enough to diagnose omental infarction, and ultrasound scan is operator dependent with a reported sensitivity of 64%, while CT scan despite having a much better sensitivity of 90% is interpreter dependent requiring experience [6, 11].

Two treatment options exist for omental infarction: conservative/medical management and surgical intervention, which is usually done laparoscopically. Currently, there is no consensus on the best treatment modality, but with increasing preoperative diagnosis, conservative treatment has gained popularity since omental infarction is seen as a self-limiting condition [2]. This approach employs analgesics, anti-inflammatory drugs, and occasionally antibiotics, and a number of case series have reported success [2, 6, 7, 11]. However, complications do occur albeit rarely with conservative management. These include prolonged/worsening pain, abscess formation, adhesions, and intestinal obstruction [7, 11].

The proponents of surgical approach argue that surgery (usually laparoscopic) expedite the resolution of symptoms and shorten hospital stay in addition to preventing the complications associated with conservative treatment [2]. However, there is no denying the risks associated with any surgical intervention.

In the rare case of omental infarction occurring simultaneously with another more serious acute abdominal pathology like appendicitis as in our patient, surgical intervention is the treatment approach of choice. Both two cases reported in literature thus far [4, 5] and our patient had appendectomy and resection of the infarcted omentum. We, as well as Koay and Mahmoud [5], did laparoscopic appendectomy and resection of the necrotic omentum while Battaglia et al. [4] did open appendectomy and resection of the omentum. In all three cases (including ours), the patients had uneventful recovery but the hospital stay was much shorter: 2 days for the two patients who had laparoscopy compared to 5 days for the patient who underwent open surgery.

Laparoscopy not only aids in the treatment of omental infarction but also can be diagnostic such as in our case where it was missed preoperatively with the radiological imaging. Had we done an open appendectomy with McBurney's incision, there is every possibility that the patient would have continued to have postoperative symptoms leading to prolonged hospital stay as well as requiring further diagnostics and intervention to manage the same. Hence, laparoscopy is clearly superior in diagnosis, management, and resultant shorter hospital stay with resolution of symptoms. Based on our experience, we recommend placement of ports as per surgeon comfort and usually can be managed without placement of additional ports than already required for the appendectomy. However, this has to do with the skill and comfort level of the surgeon; if additional ports are required for the safe resection of the infarcted omentum, they should be placed based on the location of infarcted omentum and intraoperative surgeon judgement.

## 4. Conclusion

Imaging, especially abdominal CT scan, is central to the nonoperative diagnosis of omental infarction and as such; it is imperative that both radiologists and physicians become familiar with its interpretation and have a high index of suspicion of omental infarction after ruling out more serious causes of acute abdomen. When the diagnosis is made preoperative, we recommend a trial of conservative management and only doing surgical intervention when conservative management fails or in the rare case of a complication.

For the patient with both acute appendicitis and omental infarction, we recommend laparoscopic appendectomy and resection of the infarcted omentum and only doing open resection when the expertise and/or resources for laparoscopy is lacking.
